# Understanding the hindrance factor of bacterial proliferation and γ-aminobutyric acid-producing capability of nondairy strains of *Lactiplantibacillus plantarum* in milk fermentation

**DOI:** 10.1038/s41598-023-38701-w

**Published:** 2023-07-15

**Authors:** Konlarat Phirom-on, Anuchida Po-ngern, Somchai Jaikhan, Sasiwan Sirichon, Sukanda Vichitphan, Kanit Vichitphan, Jirawan Apiraksakorn

**Affiliations:** 1 Department of Biotechnology, Faculty of Technology, Khon Kaen University, Khon Kaen, 40002 Thailand; 2 Fermentation Research Center for Value Added Agricultural Products (FerVAAP), Khon Kaen University, Khon Kaen, Thailand

**Keywords:** Biochemistry, Biotechnology, Microbiology

## Abstract

γ-aminobutyric-acid (GABA) is a mental health-supporting substance that helps release anxiety and depression and improves memory. *Lactiplantibacillus plantarum* SKKL1, a GABA-producing bacterium, has been introduced to formulate a gut-brain axis product. However, growth and sugar consumption of *L. plantarum* SKKL1 in milk were ineffective. This obstacle was investigated by varying different types of milk, sugars, fermentation temperatures, and times. The results revealed that none of these parameters improved growth and bacterial metabolism in milk, except addition of soluble protein as found in yeast extract and malt extract. Although a protease deficiency of *L. plantarum* SKKL1 was discovered, it was not a primary barrier to cell propagation. Insight of this study showed clearly that soluble protein was an essential metabolic activator for growth, nutrient consumption, and protease synthesis, then stimulated lactic acid and GABA productions. While, milk casein and casein hydrolysate, a complex protein structure with low solubility, were not utilized by *L. plantarum* SKKL1. The novelty of this study is the first in-depth investigation to confirm a significant effect of soluble protein on enrich-GABA milk fermentation by *L. plantarum* SKKL1 as the sole starter without protease and monosodium glutamate addition.

## Introduction

γ-aminobutyric acid (GABA) is a non-protein amino acid synthesized from a decarboxylation reaction. This reaction requires glutamic acid as substrate, glutamate decarboxylase (GAD) (EC 4.1.1.15) as biocatalyst, and vitamin B6 as a coenzyme in an acidic environment^1^. GABA plays an essential role as an inhibitory neurotransmitter in the central nervous system. It directly influences the personality and the stress management of mammals, resulting in relaxation of the human brain, sleep improvement, and lower blood pressure^2^. GABA is found in many kinds of plants such as tea leaves and tomatoes^3^. Moreover, it is among the bacterial metabolites of lactic acid bacteria.

Some lactic acid bacteria are potentially probiotic, which is defined as beneficial living organisms that can survive in the human digestive tract by resisting low pH, bile salts, and digestive enzymes. The presence of viable probiotics in the colon provides many benefits to the host by balancing the intestinal microflora, lowering cholesterol levels, and stimulating immunity^4^. Numerous studies have proposed that the gut is the second brain of humankind under the concept that a gut-brain microbiota axis exists as these systems possess bidirectional communication between the central and the enteric nervous system, linking emotional and cognitive centers of the brain with peripheral intestinal functions^5^.

To promote the gut-brain microbiota, GABA-producing probiotics have been implemented in fermented foods. *Lactiplantibacillus plantarum* strains SKKL1, SKKP1, and NBK10 are GABA producing probiotics were isolated from Thai traditional fermented foods^6^. However, yogurt fermentation using *L. plantarum* has faced obstacles since this bacterium is considered a non-dairy inoculum that is not able to effectively grow in milk^7–9^. Moreover, according to the regulation of food and drug administration in Thailand, *Streptococcus thermophilus* and *L. bulgaricus* are required to use as a co-culture starter for yogurt production. As a result, it limits studies regarding bacterial metabolites and production of *L. plantarum* as a sole bacterium. No comprehensive study that investigates the hindrance factors of using *L. plantarum* as a pure-culture for fermented milk has been published as many studies suggest manufacturing yogurt by co-culturing *L. plantarum* with other lactic acid bacteria such as *S. thermophilus* and *L. bulgaricus*^10–12^.

Generally, milk is a nutrient-dense source of proteins and carbon, which are essential components for bacterial metabolism and growth, making it an ideal substrate for many strains of bacteria. Furthermore, bovine milk is typically a good substrate for GABA biosynthesis because it contains approximately 9.07 mg/100 g of free glutamic acid^13^ and up to 0.52 mg/L of vitamin B6^14^. As result, supplemental addition of monosodium glutamate to milk might not be necessary, especially for functional GABA-containing food production. The findings of the current study will contribute as one aspect of supporting evidence for the hypothesis that consuming GABA-producing probiotics could establish GABA-producing bacteria in the microbiota of the gut. This would provide GABA to an individual without the need to consume GABA containing probiotic foods. In this manner, GABA would be continuously produced and available.

Therefore, to determine the primary obstacles to GABA-rich fermented milk production by *L. plantarum*, various strains of this bacterium, SKKL1, SKKP1, and NBK10, were evaluated. The type of bovine milk and fermentation parameters were investigated. The effects of sugar, protein and protease enzyme addition were examined. Examination of the capability of such bacteria to produce GABA in milk-based media was conducted.

## Results and discussion

### Growth and metabolism of *L. plantarum* in milk fermentation

*L. plantarum* SKKL1, SKKP1, and NBK10 were isolated from traditional Thai fermented foods and their probiotic properties characterized. Tolerance of low pH, bile salts and antibiotic susceptibility were examined^6,15^. These probiotic bacteria grew considerably and produced GABA in MRS containing MSG. Regardless of how well bacteria grow in MRS media, their growth in milk did not meet expectations.

Whole and skim bovine milk were compared for fermented milk production. Considering the milk composition, two hundred milliliters of whole milk contained 8 g of fat, 25 mg of cholesterol, 6 g of protein, 10 g of carbohydrate, and 85 mg of sodium. In contrast, skim milk contained 0 g of fat, less than 5 mg of cholesterol, 7 g of protein, 11 g of carbohydrate and 100 mg of sodium. The primary difference between whole and skim milk is their fat and cholesterol content.

The results showed that after 48 h of fermentation in whole and skim milk, the number of viable cells reached approximately 10 log (CFU/mL), but the pH was still higher than the yogurt criterion of 4.6^16^, as shown in Table 1. Thus, *L. plantarum* was not a suitable as a pure culture starter for yogurt making. Many studies suggested that the content of fat in milk is one of the crucial factors affecting the yogurt flavor, taste, and texture characteristics, such as hardness, springiness, and syneresis, rather than the activity of bacteria^17,18^. Yogurt is recognized as one of the most consumed fermented products worldwide. Therefore, using fermented milk is one of the best ways to introduce GABA producing bacteria into the diet. Slow fermentation was found in the NBK10 strain. It could achieve a pH of 4.3, which assured yogurt curdling. However, using 72 h for yogurt production is inefficient as this would requires high operational costs in commercial-scale production. Therefore, increasing the fermentation temperature might hasten the metabolism of the bacterial cells during fermentation.

**Table 1:** Comparison of fermented milk properties by the SKKL1, SKKP1, and NBK10 strains using skim and whole milk at 30 $^\circ$C for 72 h.

Result	Probiotic strains					
	SKKL1		SKKP1		NBK10	
Time (h)	48	72	48	72	48	72
Skim milk						
Viable cells (Log CFU/mL)	9.8	10.2	10.2	11.3	10.8	8.9
pH	5.1	4.9	5.9	5.6	5.5	5.2
Whole milk						
Viable cells (Log CFU/mL)	10.1	10.3	10.1	11.0	10.4	11.2
pH	5.5	5.3	6.1	5.7	5.1	4.3

### Effect of fermentation temperature on fermented milk properties

**Figure 1:**
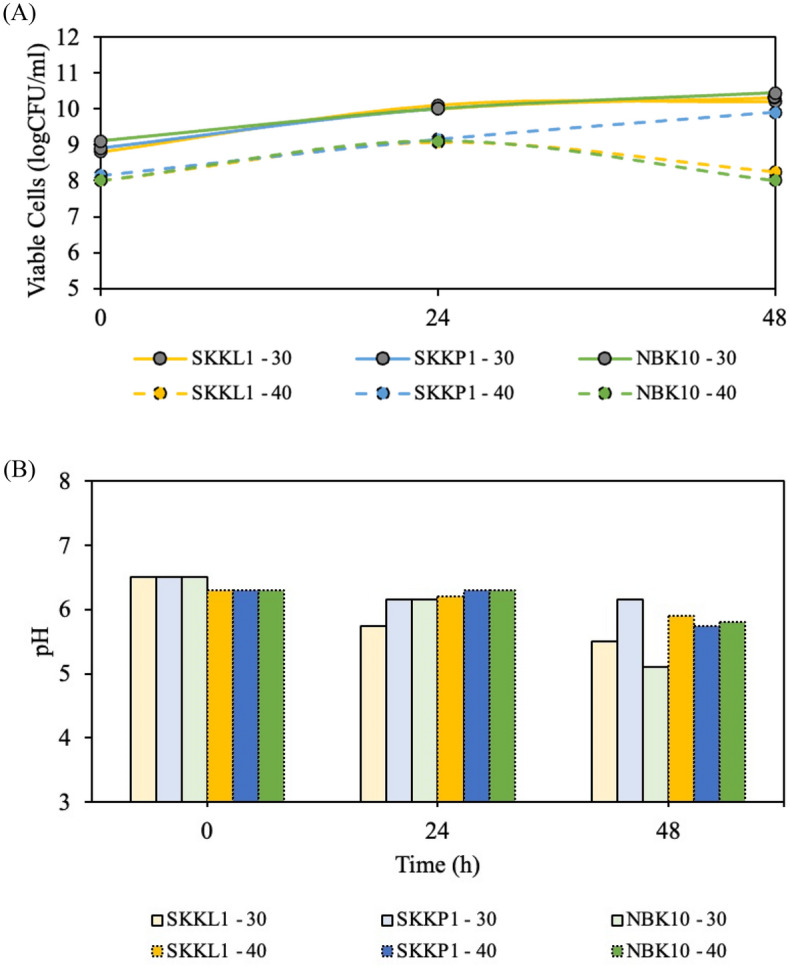
Growth curve of *L. plantarum* SKKL1, SKKP1, and NBK10 in a whole milk medium at 30 °C and 40 $^\circ$C (**A**), and resulting pH values (**B**).

The fermentation temperature was originally set at 30 °C, in which *L. plantarum* could grow slowly during the fermentation period. However, this condition could not be used for making curdle milk due to the failure to obtain a pH value below 4.6, as shown in Fig. 1B. When the temperature was increased to 40 °C, the bacterial cells were inactivated, and a decrease in the number of viable cells was observed (Fig. 1A). This agrees with the study of Shan et al.^19^ who reported that the viable cells of *L. plantarum* NDC75017 declined with incubation at 35 °C, and then were completely inactivated at 45 °C. As a result, the current study was conducted at 30 °C.

Other studies suggested using another high-temperature culture starter to create acidic conditions in milk and subsequent curd formation, followed by inoculating *L. plantarum* for nutritional and sensory improvement. Jouki et al. used a two-step method to make yogurt, first fermenting milk with *S. thermophilus* and *L. bulgaricus* at a higher temperature to achieve a low pH of 4.5, and then adding *L. plantarum*^10^. Zhang et al. employed co-culture starters using *S. thermophilus, L. bulgaricus,* and *L. plantarum* WCFS1 at 42 °C for 12 h for low-sugar yogurt production^12^. The research of Sidira et al. was also conducted with mixed starters for yogurt production^11^. As a result, it is seen that *L. plantarum* is not an ideal probiotic for yogurt production on its own. However, no report explains why *L. plantarum* produced very low acid concentrations in milk fermentation. Thus, media composition was a further focus. Only the SKKL1 strain was selected for further research, because of its thorough characterization and successful application as a potential probiotic for fermented products in other studies (unpublished data).

### The effect of glucose and lactose addition on milk properties fermented by *L. plantarum* SKKL1

**Figure 2:**
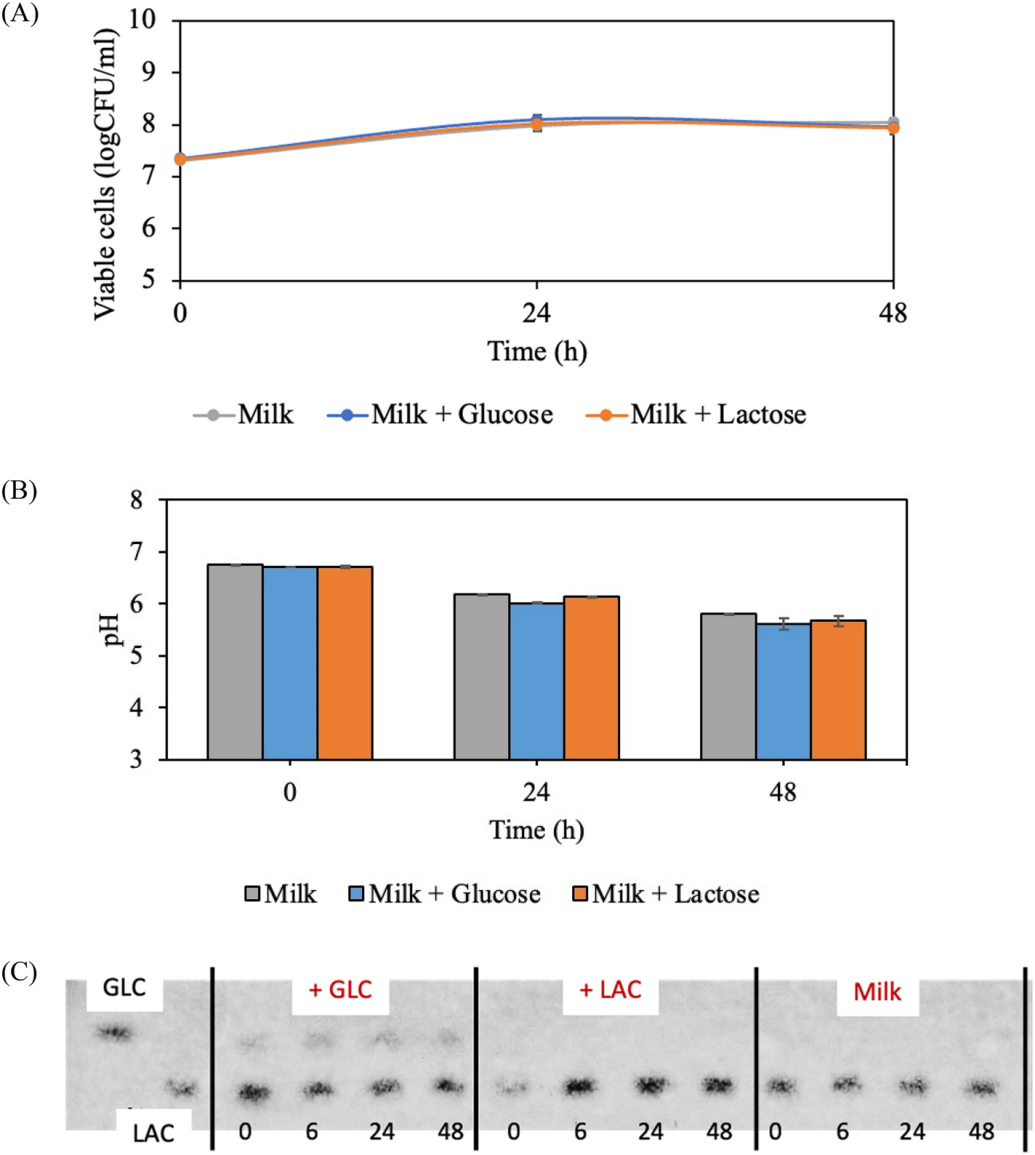
Effect of glucose and lactose on fermented milk properties (**A**) viable cells, (**B**) pH, and (**C**) sugar pattern on thin layer chromatography (TLC) plate with glucose (GLC) and lactose (LAC) as standard markers during fermentation by *L. plantarum* SKKL1 at 30 $^\circ$C for 48 h.

The findings demonstrate that viable cell counts from addition of glucose and lactose were not significantly different to that of milk with no sugar addition (Fig. 2A). Fermented milk containing glucose and lactose was characterized by a lower pH value compared to milk without added sugar, but after 48 h of fermentation (Fig. 2B). The resulting sugar content (Table 2) and sugar pattern by TLC (Fig. 2C) show low sugar consumption corresponding to poor growth by the SKKL1 strain. Glucose and lactose remained at their initial levels in all milk samples. Interestingly, the SKKL1 strain did not consume glucose when it was growing in milk. Glucose is the most prevalent monosaccharide used by lactic acid bacteria. In contrast, other studies confirm that *L. plantarum*, including the SKKL1 strain, effectively consumed lactose in modified MRS medium due to a gene encoding β-galactosidase^20,21^ to produce a high amount of lactic acid^12^. There should be another limiting factor in milk fermentation that restricts sugar uptake by this bacterium. This is because when the media composition was altered (from MRS to bovine milk), the sugar consumption of bacteria was remarkably changed. It became a viable but nonculturable bacterium, similar to one that had been exposed to environmental stress such as nutrient deprivation, heat, osmotic stress, and low pH.

**Table 2:** The levels of reducing sugars, glucose, glutamate, and GABA in fermented milk produced by the SKKL1 strain at 30 $^\circ$C after 48 h.

Results	Reducing sugars (g/L)		Glucose (g/L)		Glutamic acid (mg/L)		GABA (mg/L)	
Time (h)	0	48	0	48	0	48	0	48
Milk	54.70	52.49	ND^1^	ND^1^	133.95	57.79	ND^2^	ND^2^
2% GLC	68.10	73.00	23.70	22.50	116.91	31.22	ND^2^	ND^2^
2% LAC	69.05	67.51	ND^1^	ND^1^	85.63	40.51	ND^2^	ND^2^
1% YE	51.69	32.99	ND^1^	ND^1^	2,088.63	1,873.89	20.46	65.72
1% ME	59.23	34.00	ND^1^	ND^1^	275.83	16.31	ND^2^	ND^2^
5% ENZ	39.11	30.81	ND^1^	0.21	166.80	356.28	ND^2^	17.62
5% INENZ	36.72	27.86	ND^1^	ND^1^	180.80	75.90	ND^2^	ND^2^

*GLC* glucose-added milk, *LAC* lactose-added milk, *YE* yeast extract-added milk, *ME* malt extract-added milk, *ENZ* active protease-added milk, *INENZ* inactivated protease-added milk.

^1^*ND* not detected (< 0.125 g/L) for sugar analysis.

^2^*ND* not detected (< 2.5 mg/L) for amino analysis.

### Influences of protein supplementation on bacterial metabolism in milk fermentation

**Figure 3:**
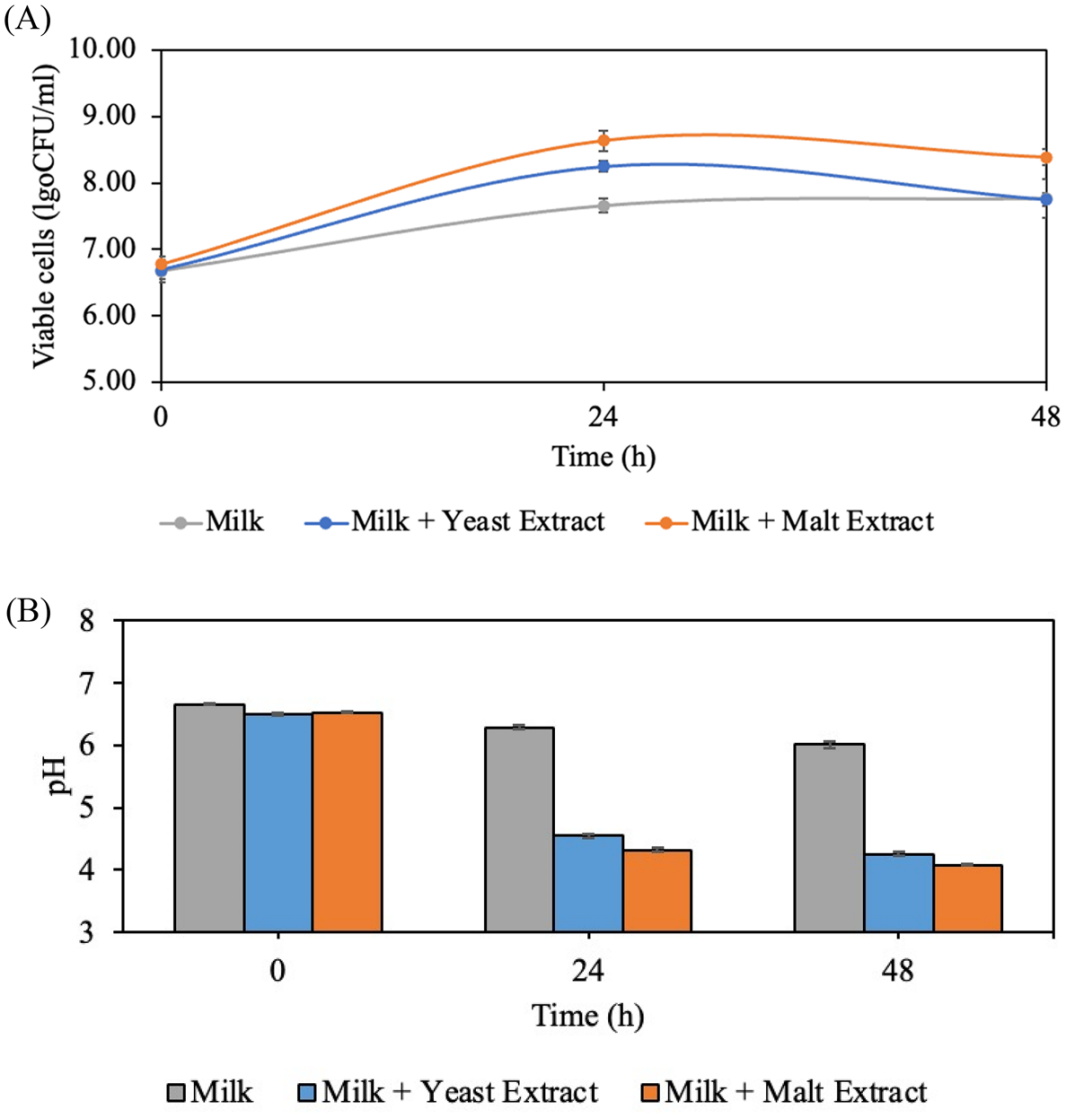
Effect of yeast extract and malt extract on fermented milk properties (**A**) viable cells, (**B**) pH during fermentation by *L. plantarum* SKKL1 at 30 $^\circ$C for 48 h.

The factor that prevents sugar consumption and bacterial metabolism might be an inappropriate nitrogen source for cell growth. The results showed that addition of yeast extract or malt extract could significantly enhance bacterial growth compared to a milk fermentation into which no protein was added (Fig. 3A). The pH of the milk samples to which yeast extract or malt extract were added eventually reached 4.5 and 4.3 within 24 h of fermentation, which is required for yogurt production, (Fig. 3B) and provided curd formation. These results agree with the study of Li et al.^22^. Milk-coagulating activity by *L. plantarum* was observed in milk to which yeast extract was added. Alternatively, the milk sample with no protein addition remained in a neutral state throughout the entire fermentation, with a pH of 6.01.

The changing content of reducing sugar confirmed that the bacteria grew remarkably better than when no protein was added. This is evidenced by the decreased sugar content, from 52 g/L and 59 g/L to 33 g/L and 34 g/L for milk samples supplement with yeast extract and malt extract, respectively. Whereas, the milk samples with no protein added showed no sugar utilization at the end of fermentation (Table 2). Sugar uptake and its metabolism start when the bacteria have a suitable protein source. This finding indicates that the SKKL1 strain could not consume sugar without suitable protein supplementation. It was previously reported that the SKKL1 strain did not grow in sugar-free MRS unless glucose was provided^23^.

Therefore, examination of the various sources of protein from yeast extract, malt extract, and bovine milk were focused on the soluble protein contents^24^, which were 303.9 mg/g_yeast extract_, 96.2 mg/g_malt extract_, and 25.7 mg/g_dry milk_, respectively. Yeast extract contained water-soluble molecules released after yeast cell disruption. Soluble peptides were the major component, which accounted for 50% of the total yeast mass, depending on the methods of cell lysis and recovery^25^, followed by free amino acids, minerals, and vitamin B6^26^. Also, plant-based proteins, like malt extract, are soluble products that are obtained from malt during mashing. It contains a majority fraction of disaccharides, with proteins, amino acids, soluble fiber, and vitamin B3^27,28^. In contrast, bovine milk is generally recognized as a protein-rich source, but it contains very low levels of soluble protein compared to yeast extract and malt extract. Most of the proteins in milk are intact casein^29^, which requires an effective protease to hydrolyze this complex into small molecules or peptides. To find the actual factor involved in protein hydrolysis during milk fermentation by SKKL1, protease detection and the influence of active protease during milk fermentation were further investigated.

### Influences of protease on fermented milk production

Soluble protein may be a key factor required to stimulate sugar consumption for bacterial growth and metabolism. The protease production capability of *L. plantarum* SKKL1 was assayed. These results indicate that the SKKL1 strain could not produce extracellular, intracellular, or cell-envelope proteases (data not shown). Thus, the factor that prevents milk fermentation by the SKKL1 strain might be the lack of casein-degrading proteases. To confirm this hypothesis, growth of the SKKL1 strain in milk was expected to increase higher levels when supplemental protease was provided.

**Figure 4:**
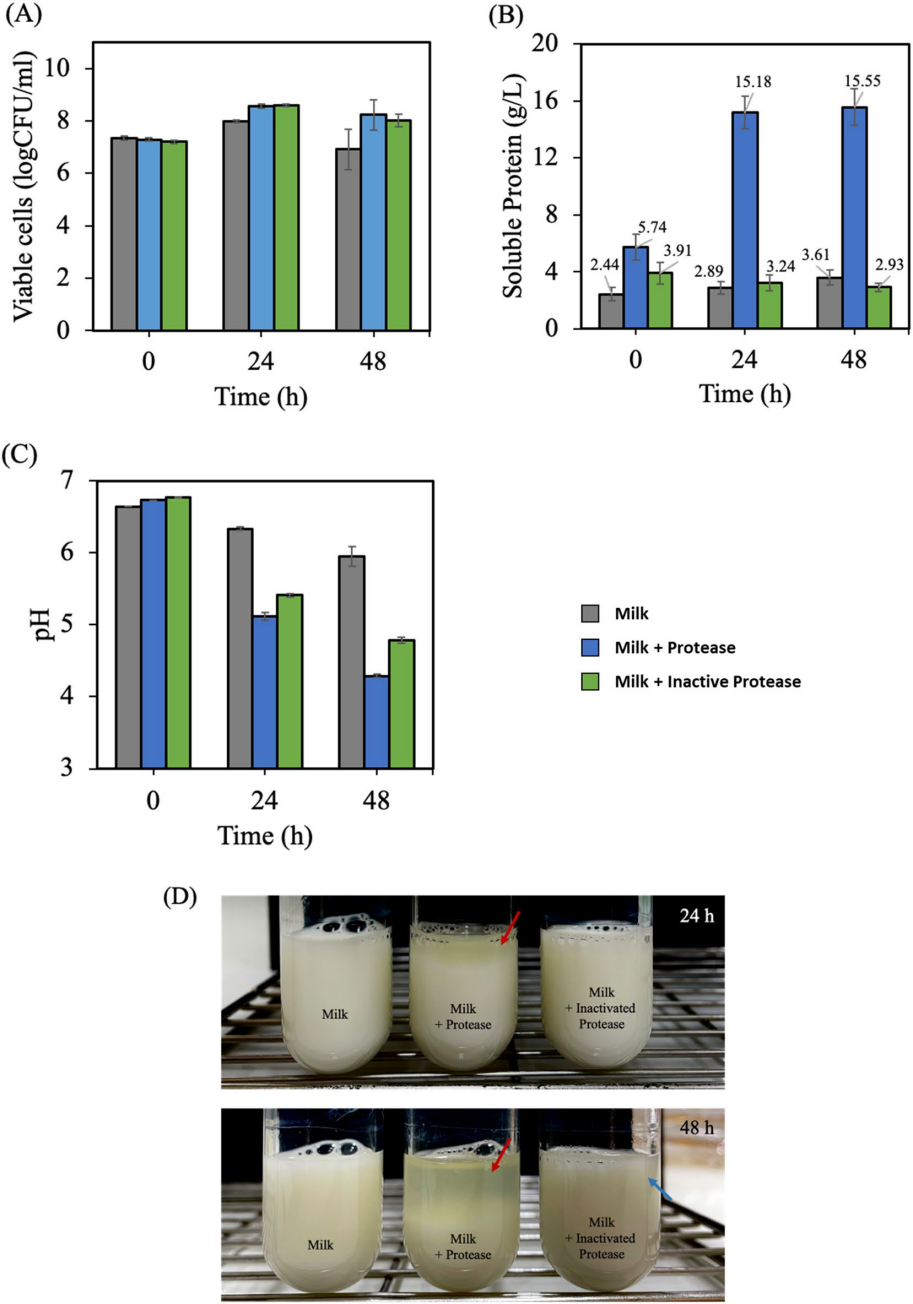
Effect of protease addition on fermented milk properties (**A**) viable cells, (**B**) the soluble protein content, (**C**) pH, and (**D**) curd-like structure of fermented milk at 30 $^\circ$C for 24 h.

The effect of protease addition on fermented milk properties is shown in Fig. 4. Addition of a crude protease enzyme could hydrolyze casein in milk and release 15.18 g/L and 15.55 g/L of soluble protein after 24 h and 48 h, respectively. These were approximately four times higher than the level of soluble protein observed when milk samples were supplemented with inactivated protease (Fig. 4B). Not only does the action of protease increase the soluble protein content, but other components in both activated and inactivated enzyme solutions also increase the soluble protein content as well. A protease enzyme was obtained from soy fermentation by *B. subtilis* J12, which originally contained hydrolyzed soy protein. Therefore, the active protease added to milk contained three different types of proteins, intact casein, hydrolyzed casein, and hydrolyzed soy peptides. The inactivated protease added to milk showed only intact casein and hydrolyzed soy peptides.

The results showed that only milk to which inactivated protease was added showed slight protein consumption since it decreased from 3.91 g/L (0 h) to 2.93 g/L after 48 h. Milk to which active protease was added provided higher soluble protein contents during fermentation. However, the bacterial growth in milk samples containing active protease and inactive protease were not significantly different. They increased from 7.20 log CFU (0 h) to 8.59 log CFU after 24 h, as shown in Fig. 4A. This finding indicates that soluble peptides released from casein protein via enzymatic hydrolysis (9.44 g/L) could not enhance bacterial growth to levels higher than that of protein content of inactivated protease (1.47 g/L). The viable cells of both samples were nearly the same at 0, 24, 48 h as seen in Fig. 4A. Surprisingly, the increase in bacterial growth was derived from protein content of the proteases (activated and inactivated forms) added to the milk samples. The soluble peptides released from casein hydrolysis did not support bacterial metabolism.

The pH values of milk samples to which active protease was added were slightly lower than that of milk samples supplement with inactivated protease (Fig. 4C). This is due to the increased free acidic amino acid content during enzymatic hydrolysis as indicated by the glutamic acid content in Table 2. Only milk samples to which protease was added met the yogurt criteria of reaching pH 4.6 after 48 h of fermentation.

The selective protein consumption of the SKKL1 strain might be influenced by a casein-targeted protease deficiency. Non-dairy strains, like SKKL1, preferentially consume simple proteins such plant proteins rather than complex protein like casein or casein hydrolysate. It could be deduced that a casein protein from animals might be problematic in microbial metabolism compared to plant-derived proteins such as soy proteins and malt extract.

### GABA biosynthesis capability in fermented milk production using probiotic nondairy strains

The content of GABA and glutamate in fermented milk using the SKKL1 strain were determined. The results showed that GABA could be found in fermented milk at 65.72 mg/L and 17.62 mg/L with the respective addition of yeast extract and protease, as shown in Table 2. The initial glutamic acid content in milk to which yeast extract was added reached 2,088.63 mg/L, which was higher than for other samples. It decreased to 1,873.89 mg/L after 48 h of fermentation. However, yeast extract initially contains 4.19 mg/g_yeast extract_ of GABA and 158.2 mg/g_yeast extract_ of glutamate as well as vitamin B6 that works as a co-enzyme for GABA synthesis^26^.

For the fermented milk samples to which protease was added, it was found that the GABA content was 17.62 mg/L. During simultaneous enzymatic hydrolysis and fermentation of milk samples to which protease was added, after 0, 24, and 48 h, the glutamic acid content increased from 166.80 mg/L to 341.86 mg/L, and 356.28 mg/L, respectively. However, considering milk samples to which inactivated protease was added, it was found that a small amount of soy protein could support the growth of the SKKL1 strain, but the glutamic acid content (180 mg/L) was insufficient to promote GABA production.

Alternatively, fermented milk samples to which malt extract was added did not contain GABA, even though the growth of the SKKL1 strain and the pH were similar to the yeast extract supplemented samples. Malt extract contains free glutamic acid (48.23 mg/g_malt extract_) and very low levels of vitamin B6, which may be inadequate for GAD activation.

When the SKKL1 strain was supplied with high amounts of glutamate and vitamin B6 in a low pH condition, glutamate decarboxylase (GAD) was activated to maintain its survival under acidic conditions. The glutamate decarboxylation system neutralizes acidic conditions in both intra- and extracellular environments by decarboxylating an acidic substrate into a neutral compound (glutamic acid, pI 3.08 to γ-aminobutyric acid, pI 7.30) and then releasing CO_2_^30^. GABA is subsequently released, resulting in alkalization of the acidic environment. As a result, a 2.6-fold increase in GABA content was observed in yeast extract supplemented fermented milk samples over a 24 to 48 h period where an acid condition existed. Hence, it can be deduced that the amount of glutamic acid, vitamin B6, glutamate decarboxylase, and favorable environmental conditions, are essential for GABA production.

## Conclusions

GABA-rich dairy products are trendy functional foods based on the gut-brain axis concept. Numerous studies attempted to produce potential probiotics with direct addition of monosodium glutamate and to address ineffective growth of *L. plantarum* in milk during a co-culture fermentation with other lactic acid bacteria. This study shows that a pure culture of *L. plantarum* SKKL1 can grow and produce lactic acid by providing an appropriate soluble protein. However, the metabolism to produce GABA was a synergism of glutamic acid derived from yeast extract or proper protease hydrolysate, and acidic conditions during fermentation process. And even *L. plantarum* currently is not on the list of approved probiotics for yogurt production, this study shows that *L. plantarum* might be a new promising candidate for yogurt production in the future. To gain insight into the metabolic behavior of *L. plantarum*, the current work proposes an approach for GABA biosynthesis in fermented milk with no monosodium glutamate addition. This GABA-rich milk product contains both GABA and GABA-producing probiotics that can assist in the continuous production of GABA under the gut-brain axis.

## Materials and methods

### Inoculum and enzyme preparation

*L. plantarum* SKKL1, SKKP1, and NBK10 were cultured in De Man, Rogosa and Sharpe (MRS) broth from HIMEDIA (consisting of 1.0% (w/v) protease peptone, 1.0% (w/v) HM peptone B, 0.5% (w/v) yeast extract, 2.0% (w/v) glucose, 0.5% (w/v) sodium acetate, 0.5% (v/v) polysorbate 80, 0.2% (w/v) dipotassium hydrogen phosphate, 0.2% (w/v) ammonium citrate, 0.02% (w/v) magnesium sulfate heptahydrate, 0.005% (w/v) manganese sulfate tetrahydrate) with 5% (w/v) monosodium glutamate) at 30 °C for 24 h. The bacterial cells were re-cultured at 18 h and then harvested by centrifugation (6750×*g* for 5 min). The cell pellet was washed twice with normal saline. The turbidity of the active cells in normal saline solution was adjusted to obtain an inoculum size of 10^8^ CFU/mL. Protease at a level of 50 U/mL was obtained from *Bacillus subtilis* J12 in soybean meal via solid-state fermentation at 37 $^\circ$C for 24 h. Heat-inactivated protease was prepared for use in a control culture.

### Investigation of factors affecting GABA production in milk fermented by *L. plantarum*

Commercially pasteurized bovine milk is generally produced as whole milk and skim milk. Both types of milk were heated to 60 °C for 30 min and cooled to 25 °C before inoculation with 10% (v/v) of the cell solution. Milk fermentation was conducted at 30 °C for 72 h. Furthermore, comparison of 30 °C and 40 °C fermentation temperatures was done. The effects of various sugars were examined by supplementation with lactose and glucose at a concentration of 2% (w/v) in milk, separately. The effects of proteins were examined by adding 1% (w/v) yeast extract and malt extract^22^, separately, to milk. The effects of sugars and protein in milk fermentation were conducted at 30 °C for 48 h. The viable cell count, pH, GABA content, sugar content and soluble protein content were monitored at 0, 24, and 48 h.

### Effect of protease on fermented milk properties fermented by *L. plantarum*

The protease and inactivated protease from *Bacillus subtilis* J12 were added to warm milk at an enzyme to substrate ratio (E:S) of 5% (v/v). Then, the milk fermentation was conducted by adding 10% (v/v) of the cell solution and incubating at 30 °C for 48 h. The viable cell count, pH, GABA content, sugar content and soluble protein content were monitored at 0, 24, and 48 h.

### Localization of protease in *L. plantarum*

The bacteria were inoculated into MRS broth and then incubated at 30 °C for 24 h. Extracellular enzymes in the cell-free supernatant were obtained after centrifugation at 6750 g$\times$ for 5 min. The cells in the pellet were lysed in an ultrasonic bath. PBS buffer (pH 7) was used to wash the intracellular enzymes. Both extra- and intracellular enzymes, as well as cell envelop enzymes were examined for protease activity^31^.

### Assays

#### Enumeration of viable cells and pH determination

The samples were tenfold diluted with 0.85% (w/v) sterile normal saline solution. Ten microliters of diluted samples were dropped-plated onto MRS agar and then incubated at 30 °C for 48 h. The colonies were counted and reported as log (CFU/mL)^32^.

The pH of samples was determined using a pH meter.

#### Sugars and sugar content determination

A qualitative sugar pattern was developed by spotting 2 µl of sample onto a thin layer chromatography (TLC) plate using silica gel 60 F254 (Merck, USA). The spots on the plate were completely dried before mobile phase movement. The mobile phase was prepared as a butanol: isopropanol: ethanol: deionized water solution in a 2:3:3:2 ratio. The TLC plates were subsequently dipped into a spot-detecting reagent prepared by dissolving 2% (w/v) orcinol in 1% (v/v) sulfuric acid in ethanol. The TLC plates were heated in an oven at 90 °C for 10 min. Then, the colors of the spots were observed. Glucose and lactose were used as standard markers^33^. The glucose content was quantified using a GOD-POD method^34^. Reducing sugar content was assessed using the DNS method^35^.

#### Determination of the GABA and soluble protein contents

Fermented milk was partially purified using 0.4% (v/v) glacial CH_3_COOH, in a 1:1 ratio of sample:acid for 12 h at 4 °C, to precipitate intact casein proteins. The clear supernatant obtained after centrifugation was filtered through a 0.45 µm syringe filter. Subsequently, the concentration of GABA was analyzed using HPLC. Briefly, an LC-20A binary pump (Shimadzu, Japan) was used with an Inersil ODS-3 column set: (4.6 × 250 mm, 5 μm) in an oven (CTO-20A) at 40 °C. The sample was analyzed using a fluorescence detector at a 350 nm excitation, 450 nm emission, × 4 gain, 5 µl flow cell, and low sensitivity^36^. The mobile phase was NaH_2_PO_4_·2H_2_O, Na-P-toluenesulfonate, deionized water, and absolute ethanol. The amino acid-detection solution was an orthophthalaldehyde (OPA) reagent containing ethanolic OPA and N-acetyl-l-cysteine in a borate buffer. The system was run at a flow rate of 0.5 mL/min of buffer and 0.2 mL/min of OPA reagent. Additionally, the clear supernatant obtained after precipitation was used to determine soluble protein levels following Lowry's method^24^.

### Protease activity

Fifty microliters of the enzyme were incubated with 350 μl of 1% (w/v) casein in a 0.1 M PBS buffer pH 7 at 37 °C for 10 min. Enzyme activity was terminated by adding 1000 μl of 10% (w/v) trichloroacetic acid solution. A sample of the clear supernatant was taken for protein determination. A control was prepared by mixing 350 μl of substate with 1000 μl of a 10% (w/v) trichloroacetic acid solution, then adding 50 μl of an enzyme solution. Increased protein content represents protease activity (modified from^37^).

## Data Availability

Correspondence and requests for materials should be addressed to J.A.
